# Latent or Manifest Observers: Two Dichotomous Approaches of Surveillance in Mental Health Nursing

**DOI:** 10.1155/2011/254041

**Published:** 2011-06-13

**Authors:** Martin Salzmann-Erikson, Henrik Eriksson

**Affiliations:** ^1^School of Health and Social Sciences, Dalarna University, S-791 88 Falun, Sweden; ^2^School of Health and Medical Sciences, Örebro University, 70182, Örebro, Sweden; ^3^Department of Acute Psychiatry, Oslo University Hospital, Ullevål Hospital, Postboks 4950 Nydalen, 0424 Oslo, Norway; ^4^School of Health, Care and Social Welfare, Mälardalen University, Box, 883, 72123 Eskilstuna, Sweden

## Abstract

*Background*. Surveillance is a central activity among mental health nursing, but it is also questioned for its therapeutic value and considered to be custodial. *Aim*. The aim of this study was to describe how mental health nurses use different approaches to observe patients in relation to the practice of surveillance in psychiatric nursing care. *Methods*. In this study, Spradley's twelve-step ethnographic method was used. *Results*. Mental health nurses use their cultural knowing to observe patients in psychiatric care in various ways. Two dichotomous approaches were identified: the latent and the manifest approach. *Discussion*. Different strategies and techniques for observing patients are structured along two dichotomies. The underlying relationships between these two different dichotomous positions transform the act of observing into surveillance. This is further developed in a theoretical model called the powerful scheme of observation and surveillance (PSOS).

## 1. Introduction

Psychiatry is closely linked to different methods of surveillance that have been used not only in relation to psychiatric patients, but also from a Foucauldian perspective [1], as a method of organizing those mechanisms of monitoring/disciplining that are applied within psychiatric clinics. As such, surveillance is one of the most crucial and central activities in mental health nursing. It serves as a mean to collect important data on patients' behaviors, which in turn informs the chosen caring strategies of nurses and physicians. For example, such data may direct whether a patient should be given pharmacological treatment or be detained under a mental health act. On the other hand, the concept of surveillance is in general a negatively loaded term. It is associated with a violation of autonomy and sovereignty due to the use of “panoptic control.” This refers to situations, where an individual supervises another individual according to a hierarchical order with the intent, to make use of the existing power that is built into this relationship. From this point of view, surveillance is still a rather undefined concept in relation to activities and assessment in mental health nursing. Further, relatively little attention has been paid to the practice of observing patients in nursing care in relation to this discussion.

## 2. Background

When reviewing the literature about mental health nursing in psychiatric care, questions concerning social control of patients emerges as a key theme. According to the most widely accepted definitions, nursing care is based on values and concepts such as “closeness”, “intimacy”, and “partnerships” with patients, which are expected to provide a point of departure for nurses' approaches and actions/thinking [2–4]. According to several researchers [5–8], psychiatric care, besides practical nursing assistance and management, also includes interventions such as “keeping an eye” on patients to reduce self harm, violence, and manipulative and suicidal behavior. Interventions related to “keeping an eye” are sometimes accused of impeding the therapeutic function nurses are to play in their patients' care. Paraphrasing Eriksson [9], who notes that nursing is motivated by a fundamental interest in safeguarding human dignity and integrity, surveillance, when perceived as a violation of autonomy and sovereignty, can clearly be debated as a source of tension in nursing practice within psychiatric settings. 

This tension derives partly from the fact that surveillance is regarded often as a restraining activity associated with modern social institutions and the utilization of technical devices. This is evident in general social arenas; for example, the National Defence Radio Establishment's ability to secretly survey information flows over the internet [10], and the increasing use of closed-circuit televisions CCTVs [11]. Institutional arenas, such as schools and nursing institutions, also use constant surveillance and monitoring [12, 13]. Moreover, Buchanan-Barker and Barker [5] show that surveillance has long been a fundamental element of mental health nursing. In fact, they argue that “observing patients” can be regarded as the origin of all mental health nursing activities. Hamilton and Manias [14] also stress that surveillance is regarded primarily as a custodial activity with no or very little therapeutic value for the patients. Buchanan-Barker and Barker metaphorically refer to psychiatric nurses' panoptic observations and viewing of patients as the “long arm of the soul doctor” [5, pages 541]. Consequently, throughout most of the reviewed literature surveillance, is connected to disciplinary power whereby patients, who are imbued with a sense that they are under constant scrutiny and observation, internalize strategies of self observation and self regulation. Holmes emphasizes that nurses throughout history and in the present have contributed to the powerful disciplinary effects of surveillance since they are an important part of this apparatus. He further argues that “it is now time to begin a reflection over and examination of the role of nurses in such settings” [7, page 14]. Moreover, in the nursing tradition, surveillance is frequently highlighted in diverse nursing contexts, including primary health care nursing [15], child health care [16], and of course in mental health nursing [7]. It is also important to bear in mind the central nursing concepts of closeness, intimacy, and partnership in relation to this context. In order to comment on and elaborate previous research on surveillance and observation, there is obviously a need for a focused understanding of the way nurses use different approaches to observe patients in relation to the practice of surveillance in psychiatric nursing care. 

## 3. Aim

The aim of this study is to describe how mental health nurses use different approaches to observe patients in relation to the practice of surveillance in psychiatric nursing care.

## 4. Methods

Since the early days of cultural and social anthropology, ethnographic fieldwork has been central to collecting data when studying cultures. For example, in the 1920s, Malinowski published his famous work from living with Trobriands in Papua New Guinea [17]. Another milestone in the development of ethnography was Goffman's [18] work of describing the social situation for patients in asylums. In the 1970's, Spradley introduced an alternative way of working with ethnographic data, illustrated in his ethnography of the culture of tramps [19] and study of culture in a college bar [20]. Leininger and McFarland [21] developed the ethnonursing research method to study transcultural care within the nursing discipline. Nowadays, ethnography has become a well-established research method in several widespread nursing contexts, such as patient council [22], nursing on an acute stroke unit [23], privacy and dignity of cancer patients [24], and nursing in a pediatric intensive care unit [25]. Ethnographic fieldwork has also been applied in the context of psychiatric care. Marangos-Frost and Wells [26] studied psychiatric nurses' feelings about the use of restraint, Hem and Heggen [27] used ethnography in order to describe nurses' rejection of psychotic patients, Johansson et al. [28] focused on encounters in a locked psychiatric ward, while Hamilton and Manias [6] conducted an ethnographic study about how psychiatric nurses used observation as an intervention in an acute psychiatric ward.

Spradley's ethnographic twelve-step Developmental Research Sequence (DRS) was used to collect and analyze data [29, 30]. In short, the following steps were taken; (1) locating an informant; (2) interviewing and observations; (3) making an ethnographic record; (4) asking descriptive questions; (5) analyzing ethnographic interviews and field notes; (6) making a domain analysis; (7) asking structural questions; (8) making a taxonomy analysis; (9) asking contrast questions; (10) making a componential analysis; (11) discovering cultural themes; (12) writing up the study.

In this study, the concept of culture is based on a definition put forth by McCurdy et al. as “*knowledge* that is learned and shared and that people use to generate behavior and interpret experience” [31, page 5]. In order to gain specific knowledge about the nursing related issue of observing patients in psychiatric settings, data was gathered and analyzed selectively. According to this focused approach, we studied “observation” from four different ethnographic standpoints: *spaces* (where were staff and when they were observing patients?), *activities* (what kinds of acts did staff engage in to observe?), *goals* (what did the staff try to accomplish by observing?), *feelings *(what kind of emotions were associated with observing?). These standpoints were not deductively analyzed but rather provided a framework for the analysis. In this study, three psychiatric intensive care units (PICU) were included and were chosen from a strategic sample of 14 PICUs in Sweden. Data were collected through ethnographic field work, which involved the first author (MSE) spending 204 hours with mental health nurses and enrolled nurses in all three wards. During the fieldwork, MSE conducted numerous informal interviews and almost 16 hours of formal interviewing. MSE engaged in the culture, going from an etic (outsider) to an emic (insider) perspective by participating in various activities, such as rounds, self-defense courses, coffee breaks, and daily idling on the wards. To accomplish this transition and become one of the group, MSE took several steps such as dressing in the same uniform as the staff, having access to keys and being able to move freely, taking part in informal conversations in the staff room, and following regular working hours and observing incidents as they took place. During the field work, questions were frequently asked about the issue of observation and interviews were recorded and field notes were taken. The data were analyzed according to the procedure described above. As hypotheses were raised during initial analyses, the researchers tested these by placing statements in interviews. For example, “In the PICU, you try to avoid the feeling of being supervised among the patients,” followed by three response alternatives, true, false, and it depends on. The statements provided a basis for further discussions in interviews. Through this abductive way of working with data, we came to a deep understanding of the activity. The field work took place over a period of fifteen months and field notes, memos, and transcribed interviews were analyzed continuously.

## 5. Ethical Considerations

Ethical approval was obtained from the Regional Ethical Review Board (no. 2008/10). Three psychiatric intensive care units from different locations in Sweden where asked to participate. After information and approval from the hospital administrative authority were received, information was given to all staff at the units. All staff agreed to participate and signed a consent form after receiving verbal and written information. Data collection and the handling of data proceeded according to appropriate rules and regulations, and as such data was made unidentified such that no individuals can be identified due to specific personal characteristics. Further, data was collected on staff members' perspectives, meaning that no data was collected about patients. 

## 6. Findings

The findings in this study are presented in relation to two separate themes: latent observer and manifest observer. These two contradictory ways of observing patients differ in the degree to which it is desirable that patients sense the presence of staff members. 

### 6.1. Latent Observer

The first observation approach was identified as the *latent observer*. This kind of approach involved the staff members being physically present in the unit, with an intention to behave as naturally and “invisibly” as possible from a patient's perspective. This approach was used in order to avoid creating negative feelings among patients, related to a sense of being supervised and monitored. During a typical shift, the staff members sat in public areas in the unit, for example, at dining tables and in sofas reading a newspaper or watching television. By being physical present in the unit, they were able to observe what happened at all times and have full control since they were in close proximity to the patients.

*It's all calm and quiet on the unit. Three people are sitting in the sofas and watching TV, one staff member and two patients. I sit down and watch TV as well. No one is saying anything. One staff member told me earlier that they observed the patients all the time, even if it is not that obvious. Another patient joins in and sits down. After ten minutes of silence, the staff member says that he is going to “take a look”. He gets up and walks away, passing all the patients' rooms and looks through the small window in the door which makes it possible to observe what is happening inside without opening the door*. *Field note from PICU2@7:30 pm. *

In this note, the staff members were not talking, but sometimes such casual moments by the TV led to informal conversations with the patients. During such moments the staff members constantly observed what happened. A program on the television or an article in the newspaper often stimulated a topic of conversation. While addressing these topics, they observed and assessed the patients' ability to concentrate and to follow along with a TV-program or how the patients reflected upon the content in the newspaper. By adopting such natural behaviors, the staff used the latent observer approach. This kind of latent approach was also used in outdoor walking situations. For example, the staff members followed patients who were not allowed to leave the unit alone to the cafeteria. On such walks, the staff talked about everyday topics while observing the patients using a latent approach. As the patient and staff members were doing something together, the staff member's role as an observer diminished, and the two partners became more equal. 

A deviation from this approach was identified. During dining or coffee sessions, the staff often stood against the wall and monitored the event, or in some cases sat at a table together with patients.

*They decide that Arne should “take the social meal” and Karin should “take the kitchen”. She sets up two trolleys. The first trolley is left inside the kitchen with the canteen and on the other trolley she places plastic plates, forks and knives in pairs, and napkins, cups in solid plastic in various colors, and two carafes with water. The trolley is placed in the dining room so the patients are able to access it, beside the dining tables. Arne waits for the patients to create a queue by the window, which is opened from the inside by Karin. She stands inside the kitchen and meets the patients through the window. She receives the plates from the patients one by one and serves food on their plates as the patients wish. Arne, who will have the social meal, also stands in the queue, as the last one. Someone asks for salt and Karin hands over a portion of salt á 1g. In this very moment, I'm asked if I want to have some food as well and I accept with gratitude. There are three dining tables with four chairs by each table, which gives a total capacity of twelve seats for a unit with eleven patients—the last seat is dedicated to the staff member who is taking the social meal. At one of the tables, Arne is sitting and diagonal to him sits a patient. I sit down next to Arne. The fourth chair at our table is left empty. I note that it is completely silent around the table. Nobody is talking. Karin is still standing inside the kitchen with the hatch open, leaning her back against the dishwasher and surveying the dining area. She is asked if she feels like the kitchen help at a school cafeteria and she replies that it is so much more silent here. And it becomes quiet again. When everybody has finished their meals, they put their plates on the trolley and throw garbage in the bag on the trolley. I do the same. Field note from PICU1@5:00 pm.*

In this example, which is one of several, the staff members were able to observe the patients capability to behave in a social event—whether they ate fast or slow, whether they were socializing or not, had an appetite or not. Another staff member also shared his observations around the dining event.

*During lunch-time, how does the patient behave when making a sandwich? Does the patient throw away the butter package or just leave it? Is there a clump of butter in the center of the sandwich? Are there fourteen cheese slices or…the small things…you see how the patients are. Interview with Regina@ PICU1.*

The staff observed how the patients behaved in a public environment and further discussed this with their colleagues and the psychiatrist. By using a latent approach, the staff members were also able to identify medical side effects, the presence of symptoms or an abatement of symptoms, and such things as escalating aggression, self-harming behavior, and anxiety. Through their relaxed approach, they observed the patients in an inconspicuous and latent manner so that the patients did not have to feel monitored. This was also seen in relation to newly admitted patients, many of whom felt disgraced by the fact that they were admitted against their will. These patients often resisted by withdrawing themselves. In such instances, the staff ensured the stability of the unit by accepting this resistance and leaving the patients alone. They were unobtrusive and waited for the patients to initiate contact. In the meanwhile, the staff inconspicuously observed such individuals as latent observers. For example, the staff members smoothly swept through the unit, from the nursing station in one part of the unit to a locker room in another part of the unit. During these sweeps, they were able to make fragment observations. By repeating this regularly, they were present most of the time in the unit but in a latent manner. 

### 6.2. Manifest Observer

The second observation approach was identified as the *manifest observer*. This approach meant that the staff members were physically present in the unit, but with an intention to make it clear to the patients that they were present and that they were observing them. This approach was often seen when the staff members had formal conversations with the patients, in contrast to the informal conversations witnessed with the latent observer approach. Sometimes the staff observed that the patients were in need of a private conversation due to anxiety or fear. In such private encounters, the staff substantiated their observations and expressed to the patient that they had identified that he or she was in need of a private talk. One example of such a situation occurred when a staff member encountered a patient who wanted to cut herself.

*I usually say “I can see that you are not well”, then the patient responds “Damn, are you sitting here and watching me” (the informant laughs a little), “Yes, we are here for your sake and want to see you”. We prefer not to say we observe you, we rather say “I can see that you are not alright”. You confirm for the patients, an affirmation, “you are not alone in your fight”. And that I understand that it feels exhausting that so many people are watching you. You are not able to be by yourself because we have our eyes on you all the time, but it is for your sake. Interview with Roland@PICU3.*

This staff member not only observed that the patient was having a hard time, but also, and more importantly, expressed the observation in order to confirm the patient's suffering. This meant that it became obvious to the patient that the staff member had observed and seen her was present and cared about her. 

Being a manifest observer is not always associated with positive feelings. When patients were physically restrained or placed in seclusion, they were under constant or intense observation. In such incidences, the staff members described the manifest observation approach as leaving them with a sense of dishonor and as someone who patients felt could not be trusted. Therefore, the latent observer approach was preferred whenever situations allowed for it. When patients demonstrated challenging or unacceptable behavior in the unit, or when patients resisted in a way that implied a risk of physical violence, the staff had to take on a manifest observer approach in order to appear strong to patients.

*Then, of course, situations develop quickly; it starts to become a riot. Then, you attend to it and demonstrate that you are a group. That is what you see when the alarm goes off. If you push the alarm, the idea is not just that many people will come to manage the patient and then leave, it is also very much about showing strength. There are a lot of people coming and then the patient surrenders. “ooh, it is not worth doing anymore”. So, it's good to have a lot of people. Interview with Mahat@PICU1.*

In such situations, the manifest approach was not used to affirm a patient's suffering, but rather to signal power in order to soothe the patient and soothe the situation.

## 7. Discussion

### 7.1. Methodological Considerations and Limitations

Reaching trustworthiness in qualitative research is widely debated [32–34]. However, using Spindler and Spindler's nine criteria of good ethnography, we would argue for the credibility of this study [35]. Throughout the study, hypotheses emerged *in situ* as memos were written down and domains emerged. The interviews were audiotaped and transcribed verbatim thereafter, while field notes, were transcribed as soon as possible. We also argue that collecting data by different means (interviewing, taking filed notes and observing) increased the credibility. The first author collected data by participating in the culture and conducted interviews and observations. Initially, the focus was broad and descriptive but as time went by, the focus was narrowed to a selective perspective. We also reflected on our own preunderstanding which might have influenced the study negatively. The first researcher had several years of experience from psychiatric care as a clinician, although the second author (HE) was able to constantly challenge interpretations as he comes from a different tradition. We argue that by using Spradley's rigor method, we were able to control and depart from our preunderstanding. The findings in this study cannot be immediately generalized, and we leave it to the reader to decide whether they may by applicable in other contexts. A limitation in this study was that no data were collected in patient rooms, although what happened in these spaces was discussed in interviews with the staff members, and follow-up questions were posed. Having said this, observing patients was one of the main nursing tasks among the staff members in the PICU and would like to further discuss this.

### 7.2. Surveillance in Mental Health Nursing

In the study, staff approaches to observing differed in individual situations and were often dependent upon the patient's problems and needs. Observing was associated with diverse techniques and two main approaches were identified, the latent and the manifest approach, as illustrated in the results.

As shown in the results, there were several reasons for observing patients, with the two most important reasons, according to the nurses, being to get to know the patients and to be aware of improvements and deterioration in the patients' mental state of being. In an informal interview, one informant explained the following: “*Even though it is very calm in the unit, you still have to be clued in, a main task is to observe*”, to emphasize the importance of this, she put her hands behind her ears and moved her head and eyes laterally (Johanna@PICU2). 

**Figure 1:**
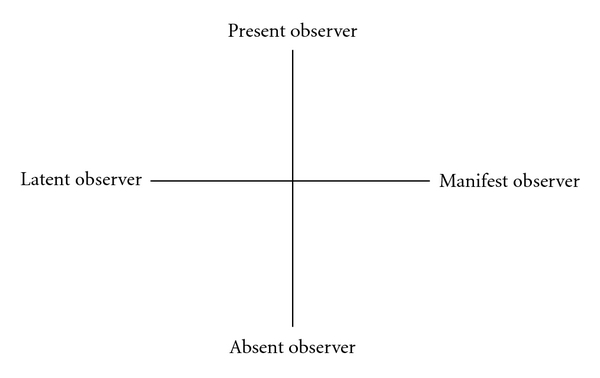
The powerful scheme of observation and surveillance (PSOS).

As shown in earlier results [5–8], the picture that emerged during the data collection was that staff members used different approaches when they observed the patients; in addition to listening and watching, they remained in close proximity to the patients, or in other situations, they kept themselves at a distance. Our findings are similar to Hamilton and Manias [6] who also identified psychiatric nurses' skills in acting discreetly as they observed patients in order to identify detailed information about the patients' behavior. In our study, it became evident that by adopting the latent approach, the staff were able to observe details. For example, they observed how a patient read the newspaper or got up from a chair, which could provide a further basis for interpretation when discussing the patient's care plan with the multidisciplinary team. The findings in this study reveal that nurses use their cultural knowing of how to approach patients in order to observe them with the overall intention to avoid provoking or arousing negative feelings around the activity of observing. In relation to results from earlier research, we can summarize these activities in Figure 1.

Different approaches are used to observe patients in order to create flexibility, which the nurses believe is “needed.” Depending on the patient and situation, different approaches offer different benefits. It is highly relevant to gain a better understanding of what is structuring pan optic surveillance in this context, and how and when specific techniques are chosen and applied. The results of this study reveal two dichotomous approaches, the latent and the manifest approach. These approaches were further structured by two additional dichotomous concepts—the present or absent observer. Using these dichotomies, we can plot the pan optic strategies for surveillance that we observed along a vertical axis indicating degrees of physical presence, and along a horizontal axis indicating a latent versus manifest approach. We would argue that nurses use cultural knowing to choose techniques along these two axes. As they do so, the relationships underlying their acts of observing transform their actions into surveillance. 

Using this four-square diagram, we argue for an extended understanding of different surveillance approaches (a) latent present, (b) manifest present, (c) latent absent, and (d) manifest absent. This is of great importance because the intersecting structure of PSOS, which impacts on the nursing care the patients receive, is largely invisible. Our attempt to shed light on this often debated, yet rarely described activity in psychiatric nursing care, offers a more nuanced understanding of observation within this setting. 

Since surveillance is such a central issue in mental health nursing, we find it very important to further explore and describe cultural knowing in relation to how nurses observe patients and which approaches are used. As we stressed in the introduction, from a Foucauldian perspective [1], surveillance and monitoring of normalcy are closely linked to each other. Neoliberal voluntary actions of (self) discipline in contemporary society can be seen in such everyday things as the use of Facebook and electronic tagging. Through the lens of surveillance, it might, therefore, be argued that the different ways of observing patients, as described in the results here, offer a way of providing nursing care that does not necessarily need to be aligned with the closeness of caring relationships. Rather, mental health nursing can include a deliberate choice to distance one's self in order to respect the vulnerability of patients. In response to patients' needs, nurses seem to shift their approaches from that of the manifest to latent observer, and vice versa. With this shift, their physical proximity to patients and the closeness of the relationship are also altered. However, both approaches are designed to allow the nurse to keep a monitoring “eye” on the patient. So, out of respect for patients' human dignity and integrity (cf. [9]), it is of great importance to understand methods of observing in mental health nursing in order to establish an alliance with the patients such that an intimate relationship can be fostered. 

## 8. Clinical Implications

Bearing in mind the need for further research in this area, we can make some initial comments on the potential implications our findings have for mental health nursing. Surveillance can violate personal integrity and be humiliating. It is essential that staff members in clinical practice become consciously aware of the two dichotomies that underlie their acts of observing patients, that is, manifest latent, present absent. Being a latent observer has the positive advantage of patients not feeling that they are being involuntarily supervised. Nevertheless, this approach might signal that the staff is uninterested, a noncaring approach. Being a manifest observer has the positive effect of providing patients with an affirmation of their suffering, yet it might also signal that the staff is too intrusive and violate personal integrity. Nurses should be aware that these paradoxes and contradictions may also offer important strategies by which they can more effectively support their patients and support themselves in their caring tasks. By understanding the positive and negative effects that these dichotomous concepts can have on a patient, we gain also a better awareness of nurses' use of observation as a method in relation to the practice of surveillance in mental health nursing. 

##  Declaration of Interest

The authors report no financial conflicts of interest.
